# *de novo* Histoid leprosy: an expatriate case recently diagnosed in Johannesburg

**DOI:** 10.1590/0037-8682-0468-2018

**Published:** 2019-12-20

**Authors:** Margareth Ann Olivier, Deepak Modi, Ozge Gunduz

**Affiliations:** 1 University of the Witwatersrand, Faculty of Health Sciences, Division of Dermatology, Johannesburg, Gauteng, South Africa.

**Keywords:** Leprosy, Histoid leprosy, Multibacillary

## Abstract

Histoid leprosy (HL) is a rare variant of lepromatous leprosy with unique clinical, histopathological, and microbiological features. A 32-year-old man from Malawi who immigrated to Johannesburg 1-year-ago, presented with a 4-month history of flesh-colored nodules on the face and trunk and hyperpigmented plaques on the chest and limbs. Skin slit smears confirmed multibacillary leprosy, and skin punch biopsies showed proliferation of spindled cells containing a large number of acid-fast bacilli. The prevalence of *de novo* HL is increasing in the era of leprosy elimination. HL cases may act as reservoirs and negatively affect the global control of leprosy.

## INTRODUCTION

Leprosy is a chronic infectious disease of the skin and nerves; it is caused by *Mycobacterium leprae*. Globally, the disease is associated with stigma and has high morbidity. Histoid Leprosy (HL) was ﬁrst described by Wade as a rare variant of lepromatous leprosy with distinct clinical and histopathological features^1^. HL may occur before or during leprosy treatment and can also occur *de novo* ^2^. The etiopathogenesis of HL is not well established, but an augmented cell-mediated and humoral immune response to localize the disease has been suggested^2^. Histoid lesions have a high bacillary load and cause a threat to the elimination of leprosy as these patients may act as reservoirs of infection. According to the World Health Organization (WHO), statistical eradication is defined as disease affliction of less than 1 per 10 000 population, which was achieved for leprosy in the year 2000^3^. The WHO data published in 2017 revealed the prevalence of leprosy as 171.948 cases worldwide (0.23/10 000)^4^. Our recently diagnosed case of *de novo* HL suggests a need for increased awareness rather than complacency towards this disease. 

## CASE REPORT

**FIGURE 1:**
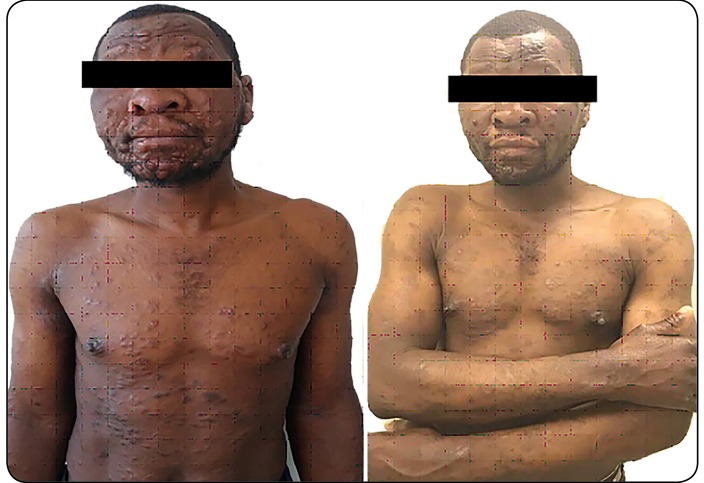
(A) Non-tender, flesh-colored papules and nodules on the face. An enlarged, infiltrated nose and scaly plaques on the body are shown. (B) One year after multibacillary-multidrug therapy.

**FIGURE 2:**
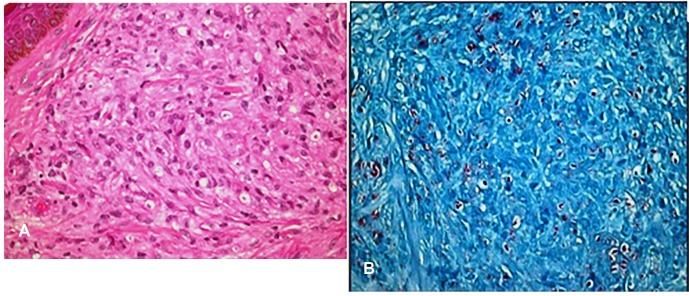
(A) The characteristic proliferation of spindled shaped histiocytes (H&E, 100×). (B) Distinct acid-fast bacilli (Modified PAS, 100×).

Our patient was a 32-year-old male who lived his entire life in Malawi. He relocated to Johannesburg for a year and reported a 4-month history of non-tender nodules on the face and trunk which had affected him socially. He had several flesh-colored papules and nodules on the face, an enlarged and infiltrated nose, and nodules on the ears (Figure 1A). There were scaly plaques and few excoriations on the chest and the limbs. He had no prior history of leprosy, leprosy contact, or any treatment for leprosy. There was bilateral peripheral nerve involvement. Skin slit smears from both the ear lobes confirmed the diagnosis as multibacillary leprosy with a high bacterial index. Biopsy taken from a nodule showed proliferation of spindle cells arranged in an intertwining pattern. The lesional skin cells had indistinct cell borders and contained large amounts of leprosy bacilli. The histological picture of clinically normal-looking skin showed areas of spindle-shaped infiltrates. PAS, Warthin Starry, and a modified Ziehl-Neelsen stain highlighted numerous acid-fast bacilli (Figure 2). These findings confirmed the diagnosis of HL. The patient showed marked clinical improvement one year after MB-MDT (multibacillary multi-drug therapy) treatment which he is continuing (Figure 1B). He developed a type 1 leprosy reaction after 8 months of treatment. Leprosy treatment was continued, and systemic steroids were added to his regimen. The reaction resolved after 3 months of therapy.

## DISCUSSION

The frequency of HL among patients with leprosy was reported to vary between 1.12−3.6%^2^ ^,^ ^5^. HL can occur even when there is no history of inadequate or irregular treatment^1^.Mathur et al. stated that the theory of inadequate WHO MB-MDT and the role of dapsone monotherapy in the development of HL are debatable as 72.2% of these patients successfully completed MB-MDT treatment, and 27.3% never received any medication^6^. The incidence of *de novo* HL is showing an increasing trend as evidenced by previous studies (summarized in Table 1). Our patient is a case of newly diagnosed *de novo* HL with no history of family or contact or previous treatment. The average age at diagnosis ranges from 21 to 40 years, and it occurs more frequently in men than in women. It presents with localized smooth shiny cutaneous and/or subcutaneous papules and nodules surrounded by normal-appearing skin. The lesions are located mainly on the trunk, hip, face, and limbs, especially on the bony prominences^1^ ^,^ ^2^. The palms and soles are usually spared. The peripheral nerves might be involved, and the ulnar nerve has been reported to be the commonest nerve involved^2^. The age, gender, localization, and morphology of the lesions of our HL case is similar to the other reports in the literature.

**TABLE 1:** Summary of previous studies on histoid leprosy.

	Freq.	***de novo***	Age	M/F	Most	Most	Nerve	L rxn.	Defor.	Tx	Relapse
		cases (%)	(years)		common localization	common presentation	invol. (%)	(%)	(%)		
Kaur (2008, India)	40/2150	12.5	37 (13−65)	5.7:1	Thighs and buttocks (67.5%)	Nodules (82.5%)	97.5	40	25	MDT	1
Mendiratta (2011, India)	11/962	54	30.7 (14−55)	4.5:1	Facial (100%)	Nodules (100%)	100	25	25	MDT	1
Nair (2013, India)	17/829	64.7	48 (28−75)	16:1	Limbs and trunk (64.7%)	Papules (100%)	100	23.5	11.76	MDT Oflo/ Mino	None
Mathur (2017, Nepal)	11/380	27.3	39.45 (21−75)	1.75:1	Upper extrem (90.9%)	Papules (90.9%)	100	18.8	None	MB-MDT	None
Canuto (2018, Brazil)	8/711	100	36.3 (21−60)	3:1	nm	nm	nm	75	37.5	MDT	nm
Our case (South Africa)		Yes	32	Male	Face, trunk, upper extremity	Nodules	Yes	Type 1	None	MB-MDT	Still under tx

**Freq:** frequency; **invol:** involvement; **L rxn:** leprosy reaction; **Defor:** deformity; **Tx:** treatment; **nm:** not mentioned; **oflo:** ofloxacin; **mino:** minocycline; **MB-MDT:** multibacillary multi-drug therapy.

Although HL is a well-established variant of multibacillary leprosy spectrum, it poses a diagnostic challenge in histopathology because it mimics many dermatological conditions because of tumoriform spindle cell proliferation, such as dermatoﬁbroma and neuroﬁbroma^1^. In a recent study, factor XIIIa (positive in dermatoﬁbromas) and S100 protein (strongly expressed by neuroﬁbromas) were suggested as markers to differentiate HL from the other tumoriform spindle cell lesions^5^. Our case was easily diagnosed because of the presence of the classical histopathological and characteristic bacterial morphology, and the presence of a high bacterial index in skin slit smears. In the literature, standard MB-MDT for a longer period^6^ or ofloxacin in combination with MB-MDT^6^ has been used for the treatment of HL with no difference in terms of relapse rate. Our case responded well, and the patient is still under treatment with long-term MB-MDT regimen.

Our patient presented with the following concerns: he had a newly diagnosed *de novo* HL, and he is a foreign born national, who lived in Johannesburg for only 1 year. This warranted a review of the incidence of leprosy in Malawi and South Africa considering the rising rate of the migrant population to South Africa into account. Estimates in 2016 showed that 1.6 million international migrants were residing in South Africa^7^, and the 2011 census reported that more than 75% of the foreign-born (international) migrants living in South Africa originated from the African continent^8^. Reports from Statistics South Africa’s 2016 Community Survey show that Malawi was among the top 5 countries of emigration to South Africa^8^. Malawi has a population of 18,622,104, and the registered prevalence rate of leprosy in 2016 was 531, of which 272 were new cases^4^. In 2015, 110 patients were reported to be on leprosy treatment in South Africa. There were 35 new cases reported in 2015, and 20 of the reported cases were foreign-born nationals^3^. We might now be witnessing an increased number of patients with leprosy, given the gradual increase of expatriates in South Africa, however, there is no available data on the prevalence of leprosy in South Africa in 2016−2017. Owing to its high mycobacterial load, *de novo* HL cases create a public health issue and require increased epidemiological surveillance and public health awareness. These cases act as reservoirs of leprosy and spread the disease even after a very good control program. Additionally, the prevalence of *de novo* cases is increasing in the era of leprosy elimination, which is also a point of concern for leprologist to research for a genetic mutation in the bacilli causing drug resistance^9^.

In conclusion, our *de novo* HL case in a foreign-born patient highlighting the increased incidence of HL in the era of leprosy elimination. This case is a point of a public health concern as HL cases have a high bacillary load, are possibly resistant to standard treatment protocols, and therefore, may act as reservoirs of infection and negatively affect the control of leprosy globally. There is a gradual increase in expatriates in South Africa. It is our responsibility as health care practitioners and dermatologists to recognize and treat these imported cases of leprosy to prevent morbidity and document and update the epidemiology of this disease. Thus identification, documentation, and management of these expatriate patients will aid the WHO’s aim to eradicate the curse of this disease globally.
